# Nosocomial Outbreak of Lassa Fever in Conakry, Guinea, 2022

**DOI:** 10.1093/infdis/jiag229

**Published:** 2026-04-23

**Authors:** Giuditta Annibaldis, Barré Soropogui, Kékoura Ifono, Jacob Camara, Mohamed Lamine Kaba, Fanta Berete, Sarah Ryter, Moussa Conde, Saa Lucien Millimono, Mette Hinrichs, Hugo Soubrier, Eugène Kolie, Nourdine Ibrahim, Bakary Sylla, Liana Eleni Kafetzopoulou, Joon Klaps, Philippe Lemey, Nils Peter Petersen, Mia Le, Emily Victoria Nelson, Kouramoudou Berete, Fodé Amara Traore, Anaïs Legand, Pierre Formenty, Fara Raymond Koundouno, Alimou Camara, Stephan Günther, Sophie Duraffour, Sanaba Boumbaly

**Affiliations:** Department of Virology, Bernhard Nocht Institute for Tropical Medicine, Hamburg, Germany; German Center for Infection Research, partner site Hamburg-Lübeck-Borstel-Riems, Hamburg, Germany; Centre de Recherche en Virologie–Laboratoire des Fièvres Hémorragiques Virales de Guinée, Conakry, Guinea; Laboratoire des Fièvres Hémorragiques Virales de Gueckédou, Direction Préfectorale de la Santé de Gueckédou, Gueckédou, Guinea; Centre de Recherche en Virologie–Laboratoire des Fièvres Hémorragiques Virales de Guinée, Conakry, Guinea; Centre de traitement épidémiologique du Camp Alpha Yaya, Conakry, Guinea; Centre de Recherche en Virologie–Laboratoire des Fièvres Hémorragiques Virales de Guinée, Conakry, Guinea; Department of Virology, Bernhard Nocht Institute for Tropical Medicine, Hamburg, Germany; German Center for Infection Research, partner site Hamburg-Lübeck-Borstel-Riems, Hamburg, Germany; Centre de Recherche en Virologie–Laboratoire des Fièvres Hémorragiques Virales de Guinée, Conakry, Guinea; Laboratoire des Fièvres Hémorragiques Virales de Gueckédou, Direction Préfectorale de la Santé de Gueckédou, Gueckédou, Guinea; Department of Virology, Bernhard Nocht Institute for Tropical Medicine, Hamburg, Germany; German Center for Infection Research, partner site Hamburg-Lübeck-Borstel-Riems, Hamburg, Germany; Department of Virology, Bernhard Nocht Institute for Tropical Medicine, Hamburg, Germany; German Center for Infection Research, partner site Hamburg-Lübeck-Borstel-Riems, Hamburg, Germany; Centre de Recherche en Virologie–Laboratoire des Fièvres Hémorragiques Virales de Guinée, Conakry, Guinea; Centre de Recherche en Virologie–Laboratoire des Fièvres Hémorragiques Virales de Guinée, Conakry, Guinea; Centre de Recherche en Virologie–Laboratoire des Fièvres Hémorragiques Virales de Guinée, Conakry, Guinea; Department of Microbiology, Immunology and Transplantation, Rega Institute, KU Leuven, Leuven, Belgium; Department of Microbiology, Immunology and Transplantation, Rega Institute, KU Leuven, Leuven, Belgium; Department of Microbiology, Immunology and Transplantation, Rega Institute, KU Leuven, Leuven, Belgium; Department of Virology, Bernhard Nocht Institute for Tropical Medicine, Hamburg, Germany; German Center for Infection Research, partner site Hamburg-Lübeck-Borstel-Riems, Hamburg, Germany; Department of Virology, Bernhard Nocht Institute for Tropical Medicine, Hamburg, Germany; German Center for Infection Research, partner site Hamburg-Lübeck-Borstel-Riems, Hamburg, Germany; Institute for Computational Systems Biology, University of Hamburg, Hamburg, Germany; Department of Virology, Bernhard Nocht Institute for Tropical Medicine, Hamburg, Germany; German Center for Infection Research, partner site Hamburg-Lübeck-Borstel-Riems, Hamburg, Germany; Laboratoire Central de Diagnostic Vétérinaire de Guinée,Conakry, Guinea; Agence Nationale de Sécurité Sanitaire, Conakry, Guinea; Department of Viral Haemorrhagic Fevers, World Health Organization,Geneva, Switzerland; Department of Viral Haemorrhagic Fevers, World Health Organization,Geneva, Switzerland; Laboratoire des Fièvres Hémorragiques Virales de Gueckédou, Direction Préfectorale de la Santé de Gueckédou, Gueckédou, Guinea; Centre de Recherche en Virologie–Laboratoire des Fièvres Hémorragiques Virales de Guinée, Conakry, Guinea; Department of Virology, Bernhard Nocht Institute for Tropical Medicine, Hamburg, Germany; German Center for Infection Research, partner site Hamburg-Lübeck-Borstel-Riems, Hamburg, Germany; Department of Virology, Bernhard Nocht Institute for Tropical Medicine, Hamburg, Germany; German Center for Infection Research, partner site Hamburg-Lübeck-Borstel-Riems, Hamburg, Germany; Centre de Recherche en Virologie–Laboratoire des Fièvres Hémorragiques Virales de Guinée, Conakry, Guinea

**Keywords:** nosocomial infection, Lassa fever, Guinea

## Abstract

**Background:**

Lassa fever is endemic in Guinea, with high seroprevalence in the forest region. However, clinical cases have been only anecdotally reported. In August 2022, a nosocomial outbreak occurred at a private clinic in the capital, Conakry, an area previously considered low risk.

**Methods:**

Suspected cases were confirmed by real-time reverse-transcription polymerase chain reaction within 24 hours. Viremia was monitored during hospitalization, and whole-genome sequencing was performed in-country within 13 days of outbreak detection. Outbreak investigation involved rodent testing in the home village of the suspected primary case.

**Results:**

Six cases were laboratory-confirmed, 5 of which were healthcare workers of the clinic. The case fatality rate was 16.7%. Viral RNA remained detectable in blood of survivors for a median of 26 days (interquartile range, 24–41 days) post–disease onset. Epidemiological investigations identified a suspected primary case, who had died of a febrile disease compatible with Lassa fever, had contact with all secondary cases, and had a travel history from Kissidougou area. Three near-complete and 1 partial Lassa virus genomes were recovered from the secondary cases, which phylogenetically clustered with genomes from central Guinea. Consistent with a common transmission source, the 4 genomes were almost identical. Rodent testing revealed a new reservoir area in eastern-central Guinea.

**Conclusions:**

This outbreak highlights the vulnerability of healthcare settings in low-prevalence areas of West Africa to nosocomial Lassa virus transmission due to human mobility. Facilitated by capacity-building programs for viral hemorrhagic fevers, rapid diagnosis, genomic analysis, and ecological assessment enabled an efficient outbreak response and control.

## Teaser

We report a nosocomial Lassa fever outbreak in a clinic in Conakry, Guinea, confirmed by molecular epidemiology, highlighting the risk of Lassa fever importation from endemic hotspots to low-risk areas within West Africa.

**(See the Editorial Commentary by Kraft and Morgan on pages 165–7.)**

Nearly 60 years after its first identification in Nigeria in 1969, Lassa fever (LF) continues to be a major public health threat. This acute viral hemorrhagic illness is caused by Lassa virus (LASV), a rodent-borne arenavirus endemic in West Africa [1]. Currently, there are no approved prophylactic or therapeutic interventions, apart from the use of ribavirin, the efficacy of which remains controversial [2].

Even if the reporting of LF cases has increased in recent years in several countries in West Africa, its real burden has still not been clearly determined [3, 4]. Transmission occurs mostly through direct contact with infected rodents or their excreta, with only limited human-to-human transmission occurring via contact with bodily fluids, particularly in healthcare settings [3]. The incubation period ranges from 2 to 21 days, with symptoms often presenting gradually as fever, general malaise, headache, and muscle pain. More severe cases may progress to bleeding, respiratory distress, shock, renal failure, neurological complications, and hearing loss [5]. However, detailed evidence of persistent viremia during LF in humans is still limited, as the majority of the data are derived from animal models [6, 7]. Death typically occurs within 10 to 14 days following symptoms onset in severe cases, with the case fatality rates (CFRs) and clinical severity varying across different regions, partly attributable to the presence of 7 distinct viral lineages [1, 8]. The disease has a clinically nonspecific presentation, with approximately 80% of infections believed to be asymptomatic or mild, making the early diagnosis and incidence estimation challenging. Lack of (differential) diagnosis, together with poor general infection prevention and control (IPC) practices, have been documented as the key factors contributing to LF human-to-human transmission in hospital-acquired contexts. Only a few nosocomial infections have been described in endemic countries, mostly in Nigeria, from the 2 hospital outbreaks in 1989 to the most recent cluster among healthcare workers (HCWs) in a tertiary health facility during the LF outbreak in 2018 [9, 10]. In 2013 in Abakaliki, Nigeria, nosocomial LF transmission occurred when a pregnant woman, managed without adequate IPC measures, infected multiple HCWs during her care [11]. In 2019, an outbreak in a nonendemic district in Sierra Leone involved several HCWs, including expatriate doctors, several of whom succumbed to the disease after delayed detection and insufficient IPC practices [12]. Outside West Africa, a total of 36 imported cases of LF in nonendemic countries occurred in the last 50 years, of which 36% were HCWs [13].

In contrast to Nigeria, which confirms hundreds of acute LF cases annually, Guinea has only anecdotally reported acute human cases since the 1980s, mostly from the forest region [14, 15]. As a consequence of the establishment of country-wide improved laboratory capacities after the outbreak of Ebola virus disease [16, 17], the frequency of case detection has increased in the last years, with >20 cases reported in the forest region [18, 19]. Seroprevalence studies performed in different areas of the country over the last 30 years have showed a high LASV seropositivity in the Faranah and N’Zérékoré regions. In contrast, LF is rarely seen in the maritime and central part of the country [20–22]. The recent identification of several acute LF cases in Faranah and N’Zérékoré regions correlates with the LASV rodent host geographical distribution, primarily circulating along the borders with Sierra Leone and Liberia [23].

Here, we report the first documented nosocomial outbreak of LF in Guinea, which occurred in August 2022. Epidemiological investigations, clinical features, laboratory findings (including genomic surveillance), and ecological studies are presented.

## MATERIALS AND METHODS

### Ethical Approval and Nagoya Permit

This descriptive research, using anonymized diagnostic surveillance data, has been approved by the National Ethics Committee of Guinea (CNERS) under the number 009/CNERS/25. This work is part of the Nagoya permit number 006/2023/PN. The collection of small mammals in the villages was performed under the authorization of the Ministry of the Environment (Ministère de l’Environnement et du Développement Durable) in the context of outbreak response investigation.

### Clinical and Epidemiological Investigations

Epidemiological and clinical data were collected from case files and through unstructured interviews. We define the index case as the first detected or reported case, and further laboratory confirmed, in the outbreak (here confirmed case 1). The primary case is defined as the first person infected in the outbreak, regardless of laboratory confirmation (here suspected case 1). A suspected case for viral hemorrhagic fevers (VHFs), as defined by the case definitions in the Guinean technical guidelines for integrated disease surveillance and response [24], is a case having VHF symptoms (fever and 2 of the following manifestations: headache, vomiting, bleeding, yellowing of the eyes, diarrhea, asthenia) together with relevant exposure history, and without laboratory confirmation. A confirmed case for VHF is a suspected case with laboratory confirmation or epidemiological link to confirmed cases or an outbreak.

### Laboratory Investigations

Ethylenediaminetetraacetic acid–blood was collected from suspected cases. Real-time reverse-transcription polymerase chain reaction (RT-PCR) was conducted at Centre de recherche en Virologie–Laboratoire des Fièvres Hémorragiques Virales de Guinée (CRV-LFHVG). The frequency for blood sampling for RT-PCR testing was at the discretion of the clinician. Viral RNA was extracted with the QIAamp viral RNA extraction kit (Qiagen, Germany) using 70 μL of the sample of interest (ie, separated human plasma or rodent organ homogenate) together with 70 µL of nuclease-free water and processed according to the manufacturer's instructions with 2 rounds of buffer AW2 washes and a 10-minute dry spin. RNA extracts were used for RT-PCR on the Rotor-Gene platform (Qiagen). Leftover RNA was stored at −20°C. For LASV diagnostics (human and rodent), the RealStar Lassa Virus RT-PCR Kit 2.0 from altona Diagnostics (Germany) was used. For differential diagnosis of human VHF suspected cases at the CRV-LFHVG, additional assays were performed to the RealStar Lassa Virus RT-PCR Kit 2.0, including the RealStar Filovirus Screen RT-PCR Kit 1.0, RealStar Yellow Fever Virus RT-PCR Kit 1.0, and RealStar Dengue Virus RT-PCR Kit 3.0. A LASV-positive RT-PCR result is defined as at least 1 of the 2 targets (S- or L-segment) being detected as per manufacturer instructions.

### Ecological Investigations

Rodents were randomly captured following the methodology as described by Pricemou and colleagues [25]. Live traps were set randomly in a total of 60 households located along the predefined transect in Douako, the area where suspected primary case originated from. Two traps were placed per household and maintained in position for 72 hours. A total of 57 rodents were then collected from the traps and sacrificed. Species were identified based on morphological features, including body measurements (head, body, tail, foot, and ear lengths) and fur color. Autopsies were performed in the field in full personal protective equipment, and blood, liver, spleen, and feces from the 57 rodents were collected in Douako and further stored at −20°C until brought back to the laboratory for further processing. Rodent organ samples were homogenized and stored in phosphate-buffered saline. Samples were pooled for viral RNA extraction and downstream molecular analysis, including RT-PCR. Positive pools were identified and further individually tested by RT-PCR and/or nanopore sequencing.

### Metagenomic Sequencing

Leftover RNA extracts or newly extracted RNAs were used for nanopore next-generation sequencing on the MinION platform (Oxford Nanopore Technologies [ONT], United Kingdom). RNA extracts and sequencing libraries were prepared as described previously [17, 26]. In brief, viral RNA was digested with DNase (TURBO DNase, Thermo Fisher Scientific), randomly reverse-transcribed, and amplified using a sequence-independent single primer amplification approach. MinION sequencing libraries were prepared using the Ligation Sequencing Kit (SQK-LSK109) according to manufacturer's instructions, loaded onto the R9.4.1 Flow Cells (FLO-MIN106D, ONT), and run on the Mk1C (ONT) device. Sequencing flow cells were reloaded with leftover libraries after 24 hours. Runs were further stopped after approximately 48 hours, and fast5 files were transferred to a laptop for basecalling and demultiplexing. In 2022, Guppy v.5.0.16 and an in-house pipeline were used to generate fastq files and consensus genomes, respectively. For this work, raw data was re-basecalled using Dorado v.0.7.2 and analyzed with an upgraded metagenomic nanopore pipeline (ViMOP v.1.0.2) [27]. The majority consensus sequence consisted of bases called at a minimum depth of 20×. The complete sequences have been submitted to GenBank under the accession numbers: PX115271-2, PV847661–PV847666 (human), and PV847667–PV847668 (rodent).

### Phylogenetic Analysis

For lineage classification using phylogenetic analysis, we aligned the newly generated sequences to a database of representative sequences from every LASV lineage using MUSCLE [28]. A maximum likelihood phylogenetic tree was constructed using the IQ-TREE 2 software, obtaining branch supports with the ultrafast bootstrap model [29]. For visualization, the Interactive Tree of Life (iTOL) was used [30].

## RESULTS

### Case Detection and Declaration of the Lassa Fever Epidemic

**Figure 1.**
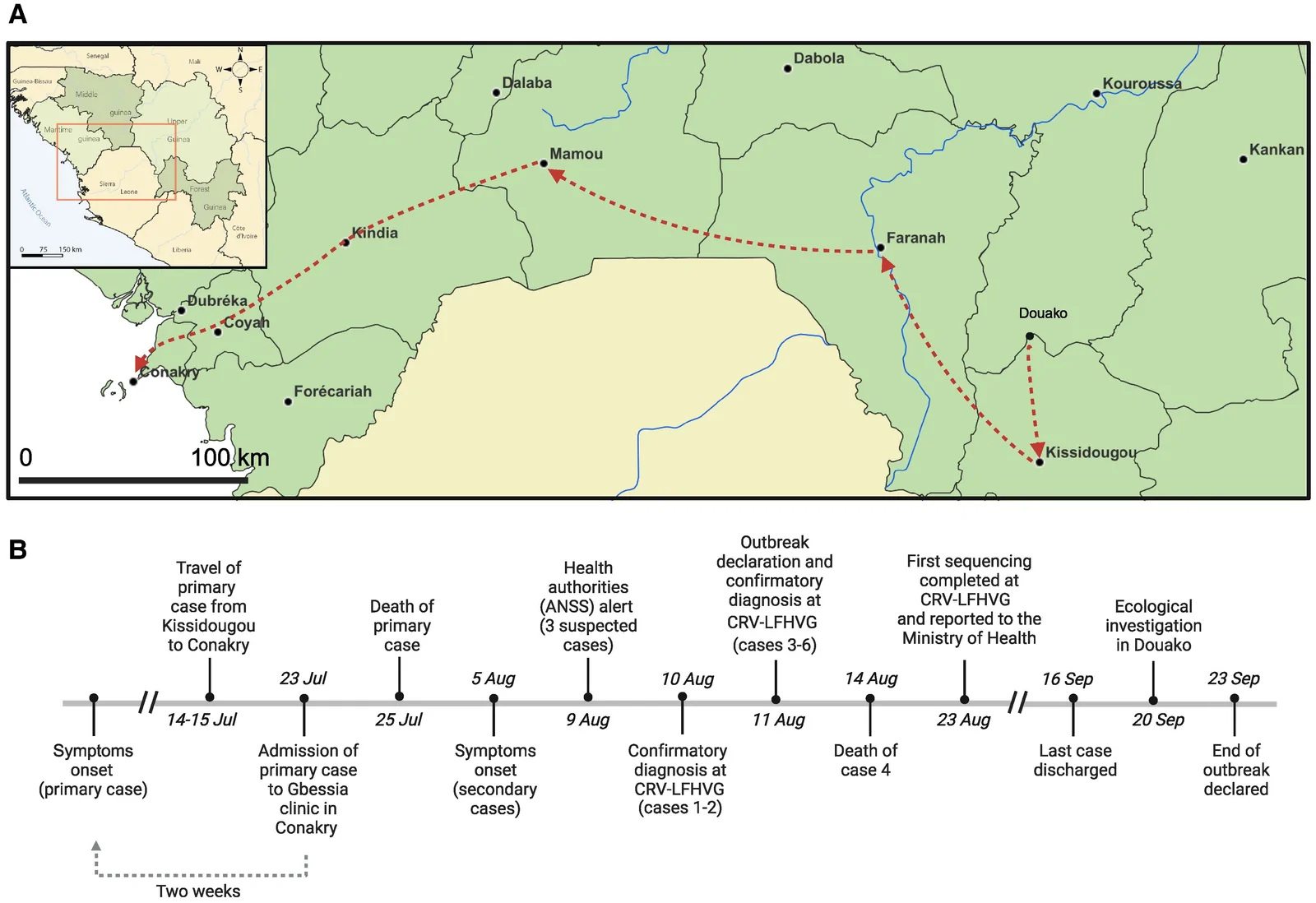
Epidemiological investigation of the nosocomial transmission of Lassa fever, with geographical history and timeline. *A*, The travel itinerary of the suspected primary Lassa fever case obtained from the epidemiological investigation is depicted. The map inset depicts a magnified region of Guinea for greater detail. The zoomed-in map traces the suspected primary case's route (red dashed arrow) from Douako to Conakry, passing through several locations, including Kissidougou and Faranah. *B*, A timeline of key events from the official declaration of the epidemic to its conclusion is summarized. Events are marked with specific dates, and findings from retrospective investigations of the case are also highlighted to contextualize the outbreak. Figure was made with BioRender (agreement number: HS29L1XVDB). Abbreviations: ANSS, Agence Nationale de Sécurité Sanitaire; CRV-LFHVG, Centre de recherche en Virologie–Laboratoire des Fièvres Hémorragiques Virales de Guinée.

On 9 August 2022, the Agence Nationale de Sécurité Sanitaire in Conakry, Guinea, received an alert from the head physician of the internal medicine and infectious diseases clinic in Gbessia of 4 suspected cases of VHF among its hospital staff. A team was immediately mobilized to conduct epidemiological investigations (Figure 1). Interviews with 3 of the 4 suspected cases (ie, 1 person was temporarily off-site and was assessed subsequently) revealed that all 3 had similar symptoms, including fever, myalgia, headache, nausea, vomiting, chest pain, and anorexia (Table 1). None of the suspected cases reported travel history in the preceding 21 days. All had been in contact with, or provided care to, a patient who died on 25 July after presenting similar symptoms along with epistaxis, hematemesis, and melena, these findings being indicative of severe systemic bleeding (Figure 1). This initial investigation supported the suspicion of VHF infection, and therefore, blood collection for laboratory testing, along with isolation of the 3 suspected cases, was done. On 9 August, their samples were sent to the national VHF reference laboratory in Conakry, the CRV-LFHVG for confirmation. Differential diagnostic testing was performed using a panel of real-time RT-PCR assays (altona Diagnostics, Germany) targeting filoviruses, Lassa virus, yellow fever virus, and dengue virus. On 10 August, LF was confirmed in 2 patients with cycle threshold (Ct) values of 30.0 and 31.2 (index case, confirmed case 1), and of 35.0 and 33.5 (confirmed case 2), for the S- and L-segments, respectively (Supplementary Table 1). Case 3 tested negative for LASV for both targets (Ct >45). The 3 patients were RT-PCR negative for all other pathogens tested. Laboratory results were immediately shared with the health authorities and in less than 48 hours after the alert, the Guinean Ministry of Health officially declared a LF epidemic. This prompted a country-wide response to investigate potential other cases and chain of transmission, as well as identify contacts to stop the spread of the disease. Equipped with nanopore-next generation sequencing capacity, on 12 August CRV-LFHVG initiated sequencing to identify the lineage and assess molecular linkage between the cases.

**Table 1.** Demographics and Clinical and Laboratory Findings of the 6 Confirmed Cases of Lassa Fever (All Employees of the Gbessia Clinic)

Case ID^a^	Age, y	Sex	Occupation	Date of First Sampling^a^	Symptoms During Hospitalization^b^	Treatment Received	Other Test Results if Applicable	Outcome	Date of Discharge or Death^a^
1	27	F	Kitchen staff	9 Aug	Vomiting, asthenia, anorexia, psychological distress	Antibiotic, anti-parasitic, anti-nausea, anxiolytic, psychological support	Malaria negative	Alive	30 Aug
2	32	F	Nurse	9 Aug	Psychological distress, no other specific symptoms reported after admission	Antibiotic, anti-parasitic, anti-nausea, opioid analgesic, psychological support	Malaria negative	Alive	31 Aug
3	55	F	Nurse	10 Aug	Cough, headache, chest pain, vomiting, asthenia, anorexia, psychological distress	Antibiotic, anti-parasitic, analgesic, anti-nausea, psychological support	Malaria negative	Alive	30 Aug
4	30	F	Nurse	10 Aug	Fever, headache, abdominal pain, vomiting, asthenia, epigastralgia; hematemesis (appeared only 24 h before death)	Analgesic, anti-nausea	Not tested	Dead	14 Aug
5	25	M	Caregiver	10 Aug	Headache, nausea, vertigo, fever, myalgia, bloody stools, tinnitus, asthenia, anorexia, psychological distress, bilateral hearing loss	Antibiotic, anti-parasitic, neuropathy treatment, anti-nausea, anti-hemorrhagic, opioid analgesic, psychological support	Malaria positive, typhoid fever positive	Alive	16 Sep
6	46	M	Doctor	11 Aug	Headache, nausea, vertigo, fever, myalgia, asthenia, anorexia, insomnia, gross hematuria, epigastralgia, bleeding (soft palate, hemorrhoids), psychological distress	Antibiotic, anti-parasitic, anti-nausea, anxiolytic, oxygen therapy, anti-hemorrhagic, blood transfusion, opioid analgesic, psychological support	Not tested	Alive	16 Sep

Abbreviations: F, female; M, male.

^a^All confirmed cases reported an identical date of onset on 5 August 2022.

^b^All patients were reported to be hemodynamically stable, excluding case 6 in the late stage of hospitalization.

### Additional Epidemiological and Laboratory Investigations

Further epidemiological investigations on 9 and 10 August suggested that the primary case (retrospectively classified as a suspected case 1) was a 30-year-old man, who died on 25 July at the Gbessia clinic, Conakry. Apart from suspected case 1, no other patients with LF-compatible illness were identified in the clinic who could be linked to the staff member's illness. A retrospective review of the patient's case file indicated that he was a community worker employed in a mining consortium from the subprefecture of Douako (Kouroussa prefecture, Kankan region), located approximately 700 km from Conakry (Figure 1). Prior to being admitted to the clinic in Gbessia, he had been sick for approximately 2 weeks and sought treatment in various clinics, likely in Kissidougou. He may have been in contact with another sick co-worker from the mining consortium, but the history could not be verified. On 14 and 15 July, he further traveled to Conakry via Mamou where he might have stopped in a clinic. The patient case file did not report a potential diagnosis or treatment he might have received during this time. On 23 July he was admitted to the clinic in Gbessia with nonspecific symptoms, including cough and fever. Laboratory testing for malaria and typhoid fever was requested; VHF infection was not suspected. The patient developed epistaxis, hematemesis, and melena on 24 and 25 July, and died on 25 July in respiratory distress. Postmortem sampling could not be performed as the body was no longer available. The primary case was classified as suspected case 1 as per national case definitions, having exhibited typical symptoms without laboratory confirmation.

**Figure 2.**
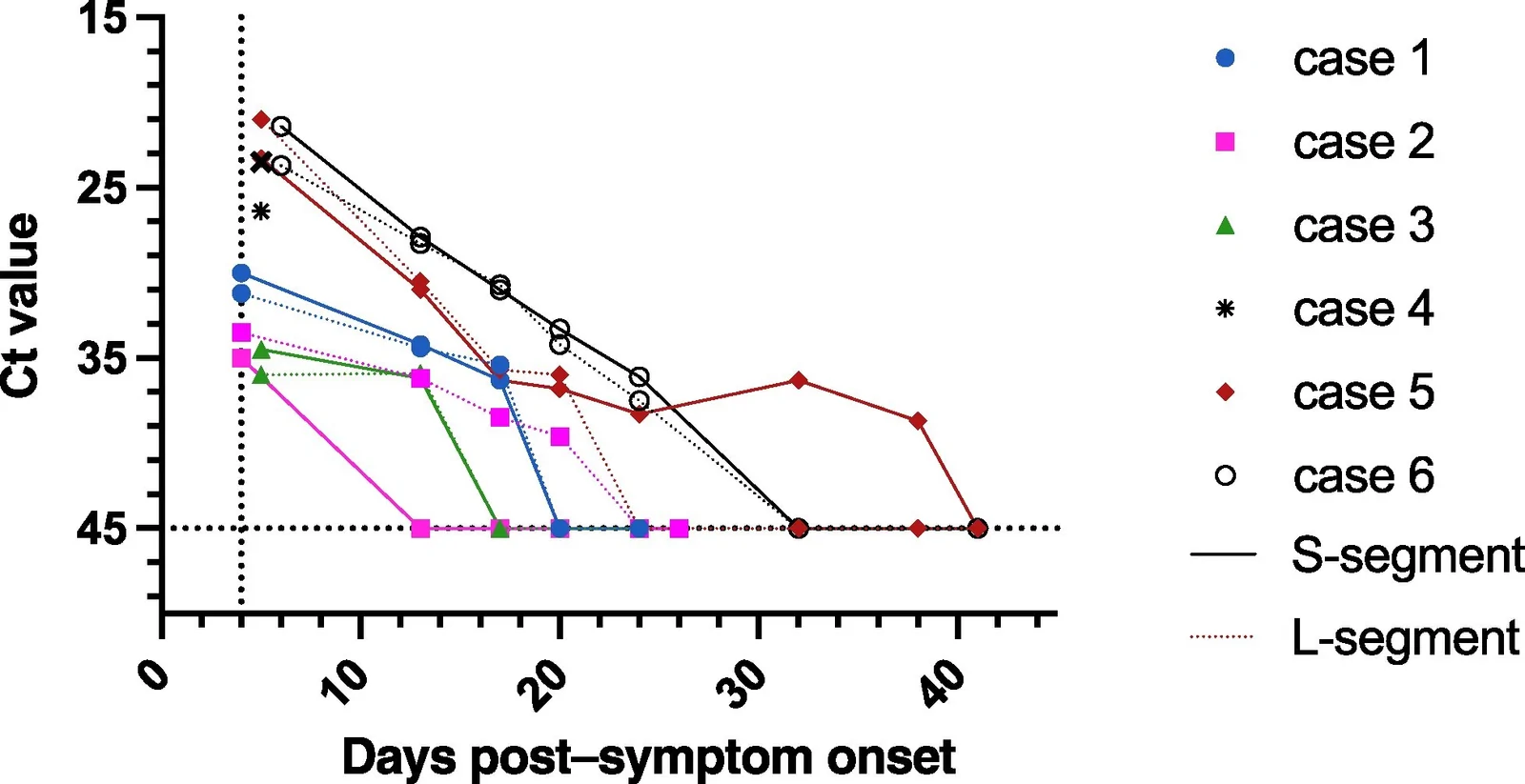
Viral load dynamics in hospitalized patients. *A*, The evolution of viral load in hospitalized patients over time is plotted using the cycle threshold (Ct) values of real-time reverse-transcription polymerase chain reaction (RT-PCR) assays. The solid line represents the S-segment of Lassa virus RNA, while the dashed line corresponds to the L-segment. Data points show the Ct values for each patient (represented by different colors and symbols), plotted over the days post–onset of symptoms. For case 4, who died on 14 August, only 1 Ct value is shown: The star corresponds to the S-segment, the crossmark to the L-segment. A Ct value of 45 indicates a result below the limit of detection of the RT-PCR test.

Further interviews at the clinic led to the identification of another 37 individuals with contact to the primary case, of which 12 were sampled on 10 and 11 August for RT-PCR testing. By 11 August, another 4 cases (cases 3–6, Figure 1 and Supplementary Table 1) were confirmed by laboratory testing, bringing the total number of confirmed cases to 6. All 6 cases were employees of the clinic, including 5 HCWs (eg, nurses, doctors, or caregivers) and 1 kitchen staff (Table 1). All reported direct contact with the primary case and an onset of symptoms on 5 August. The 6 patients were admitted to the Centre de Traitement Epidémiologique in Camp Alpha Yaya, Conakry, for clinical care. They received supportive care, including antibiotics, antiparasitics, and rehydration, and generally all were hemodynamically stable during their hospitalization (Table 1). Ribavirin was not administered due to unavailability in the country. Case 5 reported bilateral hearing loss during hospitalization and was also diagnosed with malaria and typhoid fever coinfections, prompting additional treatments. To support clinical management, viremia was regularly monitored by RT-PCR, using Ct values as a proxy for viral loads in the blood (Figure 2*A*, Supplementary Table 2). Admission of all confirmed cases (n = 6) to the ward ranged from 4 to 6 days post–date of onset with a median Ct value at admission of 28.2 (interquartile range [IQR], 22.8–34.3) for the S-segment, and of 27.5 (IQR, 22.9–34.1) for the L-segment. A steady increase of Ct values over time was observed. Time to the first negative result depends on Ct values at admission (ie, the higher the viremia, the longer the decay period; Figures 2*A*, Supplementary Table 2). Five patients survived, and 1 patient (case 4) died on 14 August, resulting in a CFR of 16.7%. Surviving patients were discharged from the ward after a median stay of 26 days (IQR, 24–41 days; n = 5) post–symptom onset. Patients were discharged based on clinical recovery assessed by the medical team combined with 1 or 2 negative LASV RT-PCR tests (Figure 2*A*, Supplementary Table 2). By 16 September, all 5 patients were discharged. From 12 August to 2 September, another 53 staff of the clinic were tested. No additional cases were detected, and the epidemic was declared over after 42 days on 23 September.

### Lineage Identification and Virus Diversity in the Confirmed Cases

**Figure 3.**
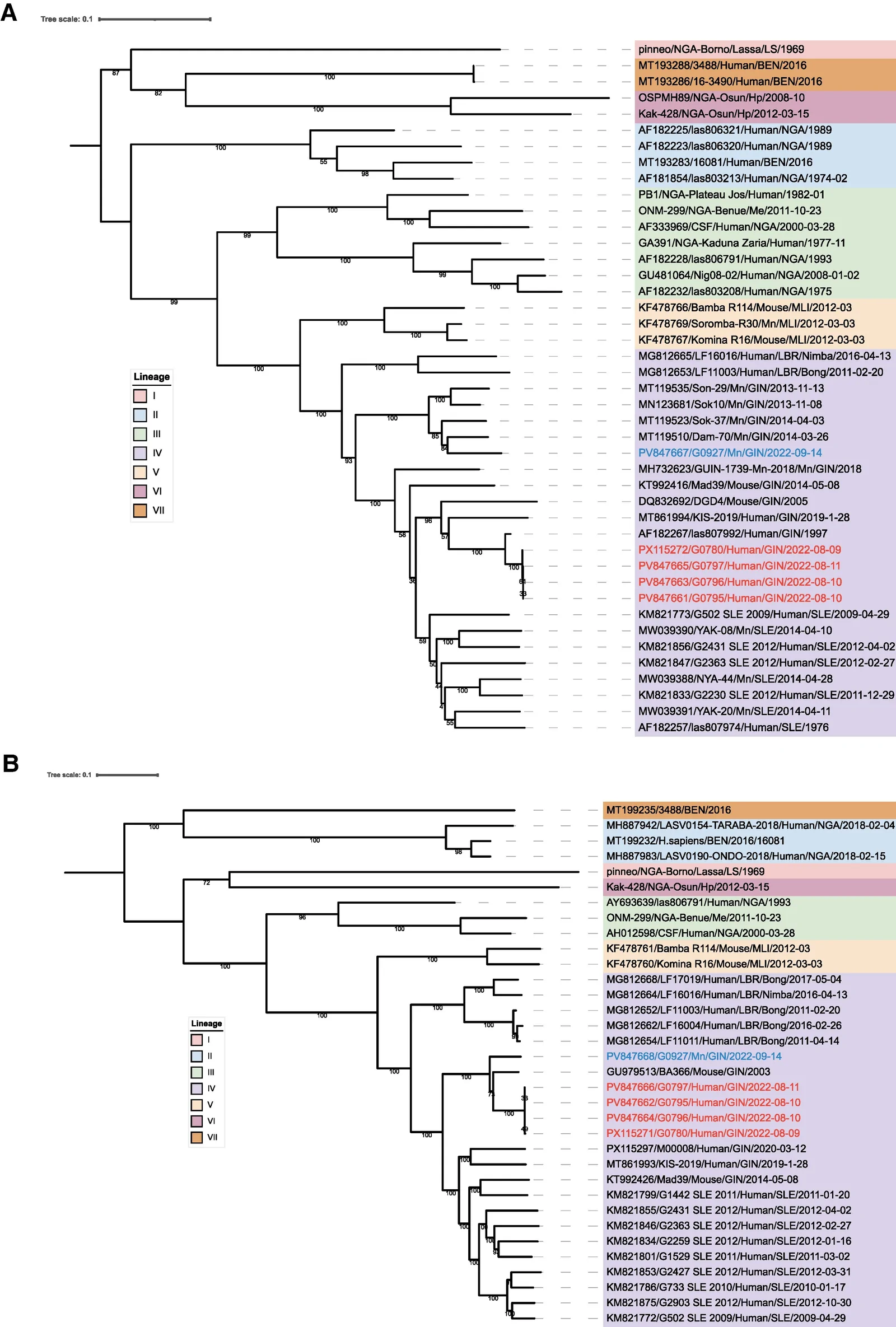
Phylogenetic analysis of the Lassa virus S-segment and L-segment from the consensus genomes of the human cases and rodent. Phylogenetic tree for the S- and L-segments of Lassa virus of the human and rodent cases detected in 2022 in Guinea. Each of the 7 Lassa virus lineages is indicated by a different color-coded background. Lineage IV is highlighted with a violet background. *A*, Sequences from S-segment obtained from cases 1 (PX115272) and 4–6 (PV847661–5) from the nosocomial outbreak that occurred in 2022 in Conakry (represented by red text), cluster with previously identified sequences from lineage IV, specifically from the prefecture of Gueckédou (DQ832692) and Kissidougou (MT861994; AF182267). The sequences cluster together without genetic differences, with 99.97% to 100.00% similarity. The obtained rodent sequence (marked in blue text, PV847667), from an ecological investigation in Douako, clusters with earlier sequences from lineage IV originating from the prefectures of Faranah (MT119510; MT119535) and Beyla (MT119523; MN123681). *B*, As in *A*, sequences from L-segment obtained from human cases 1 (PX115271) and 4–6 (PV847662–6) from the nosocomial outbreak (in red text) are either nearly identical or completely the same. All of the L-segment sequences, included the one from rodent 1 trapped in Douako (marked in blue text), cluster with previously identified sequence from lineage IV, specifically from the prefecture of Faranah (GU979513), Guinea.

In-country metagenomic nanopore sequencing was performed immediately following laboratory confirmation, providing a preliminary lineage IV identification on 19 August. Further efforts yielded partial (case 1, with S- and L-segment recovery of 36% and 42%, respectively) to near complete (cases 4, 5, and 6, ranging from 95% to 98% for the S-segment, and 95% to 100% for the L-segment) LASV consensus sequences for 4 of the 6 confirmed cases (Figures 2*B*, Supplementary Table 1). Several attempts to recover consensus genomes from cases 2 and 3 remained unsuccessful. The S- and L-segments clustered together with sequences of lineage IV previously isolated from both humans and rodents from various areas in Guinea, including Guéckédou, Kissidougou, and Faranah (Figure 3). The 3 nearly complete sequences harbored 1 and 2 single-nucleotide polymorphisms, in the S- and L-segments respectively, demonstrating a high percentage of identity between the viruses ranging from 99.97% to 100.00%, strongly suggesting a single source of transmission (Supplementary Table 3). Phylogenetic results, performed in-country, were shared in a timely manner with the Ministry of Health on 23 August and 20 September, facilitating a fast and timely response to the outbreak.

### Ecological Investigations

The primary case (suspected case 1) was from Douako, prefecture of Kouroussa, Kankan region, an area previously unrecognized for LASV circulation. His sickness duration raised the hypothesis of infection from that region. In addition, a potential virus exposure in the Kissidougou area, known to be endemic for LF, could not be excluded, but the lack of information about his place of stay or the clinic(s) he might have visited prevented further investigation. Thus, the Ministry of Health mandated a multidisciplinary team to survey rodents from Douako for LASV circulation, and, if identified, to research any linkage with the ongoing outbreak via molecular characterization. After community engagement activities were performed and local authority approval was received, the investigation team was deployed on 20 September. A total of 57 rodents were captured (29 males and 28 females). Of those, 55 rodents were identified as *Mastomys natalensis*, 1 as *Praomys rostratus*, and 1 as *Rattus*. Animals were sacrificed, and blood and organs were collected. Blood samples were pooled and tested by RT-PCR, with positive pools further leading to the identification of 4 *Mastomys natalensis* positive for LASV (Supplementary Table 4). The liver, spleen, and feces from these same rodents also tested positive for LASV (Supplementary Table 4). Metagenomic sequencing of the 4 positive blood samples allowed for the recovery of 1 consensus genome from rodent 1 with 97% of the S-segment and 94% of the L-segment covered. Phylogenetic reconstruction showed that the LASV sequence of *M natalensis* 1 belonged to lineage IV and subclustered with sequences from the Faranah region (Figure 3). No molecular linkage between LASV from the positive *M natalensis* from Douako and the human cases were observed. The sequences exhibit a divergence of 16% and 10% for the S- and L-segments, respectively. This level of variation is substantially higher than the expected mutation rate previously calculated for a consistent transmission chain of LASV [26]. Although positive rodents were found in the suspected primary case's hometown, the clustering of the outbreak sequences with previous sequences originating from the Kissidougou area suggests that the infection was acquired in the Kissidougou area, consistent with the travel history of the primary case.

## DISCUSSION

Here, we describe the first documented nosocomial outbreak of LF in Guinea. The molecular epidemiology efforts support the epidemiological investigations in confirming a single source of infection, likely the suspected primary case. Nosocomial transmission by the suspected primary case led to a superspreading event with 6 confirmed cases employed at the same clinic, of which 5 were HCWs. The prompt alert raised by the clinic (4 days post–disease onset), the rapid intervention of the investigation team from the Ministry of Health, and the available in-country diagnostic capabilities for VHFs, were essential in timely detecting the etiologic agent of the outbreak and in containing it. Considering the low-resource settings of Guinea, this reveals the critical value of local preparedness, rapid diagnostic capacity, and coordinated response mechanisms in effectively managing high-consequence infectious disease threats, as previously experienced [31]. The CFR of 16.7% observed here was notably lower than the 30% to 65% CFRs reported in previous nosocomial outbreaks in Nigeria and Sierra Leone [10–12]. However, interpretation of the observed CFR is limited by the small sample size and the absence of systematically collected clinical data, including assessment of potential coinfections.

This report also presents the first longitudinal analysis of viremia for LASV lineage IV, addressing a gap in the understanding of viral dynamics. To date, only a limited number of LF patients from Nigeria (lineage II) and few imported cases from West Africa have been studied in such details [6, 32]. Additionally, the integration of the One Health approach through ecological investigations carried out in eastern-central Guinea allowed for the detection of rodents testing positive for LASV in a new area.

Phylogenetic analysis, enabled by recently established in-country nanopore sequencing capacity, indicates that all the confirmed LF cases originated from a common source. In agreement with travel history of the suspected primary case, phylogenetic analysis suggests that infection originated from Kissidougou, which is a known LF-endemic area. However, this investigation was limited by nonsystematic data collection, incomplete documentation of the primary case's healthcare-seeking pathway, and outbreak-related IPC practices, which restricted comprehensive reconstruction of potential exposures. Nevertheless, this outbreak underscores the importance to strengthen early clinical suspicion of LF outside traditionally recognized endemic areas and to ensure that healthcare facilities are equipped to promptly implement VHF-appropriate IPC measures.

Ultimately, human mobility has long been established as a critical factor in the spread of infectious diseases, particularly in regions where endemic viruses, such as Ebola viruses and Lassa virus, circulate [16]. This report provides evidence that the introduction of LF into Conakry, a previously unaffected area, results from the movement of an individual from an endemic to a nonendemic region. The outbreak in Conakry underscores how quickly pathogens can be transmitted across regions and countries, and thus the need for effective surveillance and ready response systems.

In conclusion, this report demonstrates once more the relevance of establishing and sustaining state-of-the-art laboratory capacities for VHFs throughout the country for effectively managing epidemics [16, 17]. In addition, our findings highlight the need to reinforce LF awareness among healthcare workers and to ensure consistent application of standard IPC practices across both LF-endemic and nonendemic areas.

## Supplementary Material

jiag229_Supplementary_Data

## Supplementary Data

Supplementary materials are available at *The Journal of Infectious Diseases* online (http://jid.oxfordjournals.org/). Supplementary materials consist of data provided by the author that are published to benefit the reader. The posted materials are not copyedited. The contents of all supplementary data are the sole responsibility of the authors. Questions or messages regarding errors should be addressed to the author.

## References

[1] Garry RF . Lassa fever—the road ahead. Nat Rev Microbiol 2023; 21:87–96. 36097163 10.1038/s41579-022-00789-8 PMC9466315

[2] Moore KA, Ostrowsky JT, Mehr AJ, et al Lassa fever research priorities: towards effective medical countermeasures by the end of the decade. Lancet Infect Dis 2024; 24:e696–706. 38964363 10.1016/S1473-3099(24)00229-9

[3] Lo Iacono G, Cunningham AA, Fichet-Calvet E, et al Using modelling to disentangle the relative contributions of zoonotic and anthroponotic transmission: the case of Lassa fever. PLoS Negl Trop Dis 2015; 9:e3398. 25569707 10.1371/journal.pntd.0003398 PMC4288732

[4] McCormick JB, Webb PA, Krebs JW, Johnson KM, Smith ES. A prospective study of the epidemiology and ecology of Lassa fever. J Infect Dis 1987; 155:437–44. 3805771 10.1093/infdis/155.3.437

[5] Duvignaud A, Jaspard M, Etafo IC, et al Lassa fever outcomes and prognostic factors in Nigeria (LASCOPE): a prospective cohort study. Lancet Glob Health 2021; 9:e469–78. 33740408 10.1016/S2214-109X(20)30518-0 PMC7970450

[6] Schmitz H, Köhler B, Laue T, et al Monitoring of clinical and laboratory data in two cases of imported Lassa fever. Microbes Infect 2002; 4:43–50. 11825774 10.1016/s1286-4579(01)01508-8

[7] Thielebein A, Ighodalo Y, Taju A, et al Virus persistence after recovery from acute Lassa fever in Nigeria: a 2-year interim analysis of a prospective longitudinal cohort study. Lancet Microbe 2022; 3:e32–40. 35544114 10.1016/S2666-5247(21)00178-6

[8] Doohan P, Jorgensen D, Naidoo TM, et al Lassa fever outbreaks, mathematical models, and disease parameters: a systematic review and meta-analysis. Lancet Glob Health 2024; 12:e1962–72. 39577970 10.1016/S2214-109X(24)00379-6

[9] Dan-Nwafor CC, Ipadeola O, Smout E, et al A cluster of nosocomial Lassa fever cases in a tertiary health facility in Nigeria: description and lessons learned, 2018. Int J Infect Dis 2019; 83:88–94. 30930184 10.1016/j.ijid.2019.03.030

[10] Fisher-Hoch SP, Tomori O, Nasidi A, et al Review of cases of nosocomial Lassa fever in Nigeria: the high price of poor medical practice. BMJ 1995; 311:857–9. 7580496 10.1136/bmj.311.7009.857 PMC2550858

[11] Ajayi NA, Nwigwe CG, Azuogu BN, et al Containing a Lassa fever epidemic in a resource-limited setting: outbreak description and lessons learned from Abakaliki, Nigeria (January-March 2012). Int J Infect Dis 2013; 17:e1011–6. 23871405 10.1016/j.ijid.2013.05.015

[12] Njuguna C, Vandi M, Liyosi E, et al A challenging response to a Lassa fever outbreak in a non endemic area of Sierra Leone in 2019 with export of cases to the Netherlands. Int J Infect Dis 2022; 117:295–301. 35167968 10.1016/j.ijid.2022.02.020 PMC8948091

[13] Wolf T, Ellwanger R, Goetsch U, Wetzstein N, Gottschalk R. Fifty years of imported Lassa fever: a systematic review of primary and secondary cases. J Travel Med 2020; 27:taaa035. 32219400 10.1093/jtm/taaa035

[14] Bausch DG, Demby AH, Coulibaly M, et al Lassa fever in Guinea: I. Epidemiology of human disease and clinical observations. Vector Borne Zoonotic Dis 2001; 1:269–81. 12653127 10.1089/15303660160025903

[15] Knobloch J, McCormick JB, Webb PA, Dietrich M, Schumacher HH, Dennis E. Clinical observations in 42 patients with Lassa fever. Tropenmed Parasitol 1980; 31:389–98. 7233535

[16] Keita AK, Koundouno FR, Faye M, et al Resurgence of Ebola virus in 2021 in Guinea suggests a new paradigm for outbreaks. Nature 2021; 597:539–43. 34526718 10.1038/s41586-021-03901-9

[17] Koundouno FR, Kafetzopoulou LE, Faye M, et al Detection of Marburg virus disease in Guinea. N Engl J Med 2022; 386:2528–30. 35767445 10.1056/NEJMc2120183 PMC7613962

[18] Keita M, Cherif MS, Sivahera B, et al Case report: COVID-19 and Lassa fever coinfection in an Ebola suspected patient in Guinea. Am J Trop Med Hyg 2022; 106:1094–7. 35213814 10.4269/ajtmh.21-0713 PMC8991363

[19] Magassouba N, Koivogui E, Conde S, et al A sporadic and lethal Lassa fever case in forest Guinea, 2019. Viruses 2020; 12:1062. 32977629 10.3390/v12101062 PMC7598168

[20] Kernéis S, Koivogui L, Magassouba N, et al Prevalence and risk factors of Lassa seropositivity in inhabitants of the forest region of Guinea: a cross-sectional study. PLoS Negl Trop Dis 2009; 3:e548. 19924222 10.1371/journal.pntd.0000548 PMC2771900

[21] Lukashevich IS, Clegg JCS, Sidibe K. Lassa virus activity in Guinea: distribution of human antiviral antibody defined using enzyme-linked immunosorbent assay with recombinant antigen. J Med Virol 1993; 40:210–7. 8355019 10.1002/jmv.1890400308

[22] Marien J, Nuismer SL, Magassouba N, et al Serosurveillance identifies an endemic hotspot of Lassa fever in Faranah, Upper Guinea. J Infect Dis 2025; 232:e830–8. 40509999 10.1093/infdis/jiaf308 PMC12614985

[23] Bangura U, Davis C, Lamin J, et al Spatio-temporal spread of Lassa virus and a new rodent host in the Mano River Union area, West Africa. Emerg Microbes Infect 2024; 13:2290834. 38047354 10.1080/22221751.2023.2290834 PMC10919312

[24] Agence Nationale Sécurité Sanitaire, Ministère de la Santé et de l'Hygiène Publique de Guinée, Organisation Mondiale de la Santé Afrique . Guide technique pour la surveillance intégrée des maladies et la riposte (SIMR). Guinea, 2019.

[25] Pricemou S, Soropogui B, Bérété F, et al Diversity of *Leptospira* species and their rodent reservoirs in the Guinean forest. Microorganisms 2025; 13:833. 40284669 10.3390/microorganisms13040833 PMC12029326

[26] Kafetzopoulou LE, Pullan ST, Lemey P, et al Metagenomic sequencing at the epicenter of the Nigeria 2018 Lassa fever outbreak. Science 2019; 363:74–7. 30606844 10.1126/science.aau9343 PMC6855379

[27] Petersen NP, Le M, Renevey A, et al ViMOP: a user-friendly and field-applicable pipeline for untargeted viral genome nanopore sequencing. Bioinformatics 2026; 42:btaf687. 41460182 10.1093/bioinformatics/btaf687 PMC12809542

[28] Edgar RC . MUSCLE: multiple sequence alignment with high accuracy and high throughput. Nucleic Acids Res 2004; 32:1792–7. 15034147 10.1093/nar/gkh340 PMC390337

[29] Minh BQ, Schmidt HA, Chernomor O, et al IQ-TREE 2: new models and efficient methods for phylogenetic inference in the genomic era. Mol Biol Evol 2020; 37:1530–4. 32011700 10.1093/molbev/msaa015 PMC7182206

[30] Letunic I, Bork P. Interactive Tree of Life (iTOL) v6: recent updates to the phylogenetic tree display and annotation tool. Nucleic Acids Res 2024; 52:W78–W82. 38613393 10.1093/nar/gkae268 PMC11223838

[31] Keita M, Talisuna A, Chamla D, et al Investing in preparedness for rapid detection and control of epidemics: analysis of health system reforms and their effect on 2021 Ebola virus disease epidemic response in Guinea. BMJ Glob Health 2023; 8:e010984. 10.1136/bmjgh-2022-010984 PMC9815045 36599498

[32] Ogbaini-Emovon E, Akpede G, Okogbenin S, et al Virus load kinetics in Lassa fever patients treated with ribavirin: a retrospective cohort study from southern Nigeria. Open Forum Infect Dis 2024; 11:ofae575. 39450398 10.1093/ofid/ofae575 PMC11500659

