# Supplementary material for: Nosocomial Outbreak of Lassa Fever in Conakry, Guinea, 2022
Source: J Infect Dis. 2026 Apr 23;234(2):189–97. doi: 10.1093/infdis/jiag229 (PMC13537155; doi:10.1093/infdis/jiag229)
Supplement: Supplementary file 1 [file jiag229_supplementary_data.docx]

**Supplementary file**

**Title**

Nosocomial outbreak of Lassa fever in Conakry, Guinea, 2022

**Authors**

Giuditta Annibaldis ^1,2,^*^,§^, Barré Soropogui ^3,^*, Kékoura Ifono ^4,^*, Jacob Camara ^3,^*, Mohamed Lamine Kaba ^5^, Fanta Berete ^3^, Sarah Ryter ^1,2^, Moussa Conde ^3^, Saa Lucien Millimono ^4^, Mette Hinrichs ^1,2^, Hugo Soubrier ^1,2^, Eugène Kolie ^3^, Nourdine Ibrahim ^3^, Bakary Sylla ^3^, Liana Eleni Kafetzopoulou ^6^, Joon Klaps ^6^, Philippe Lemey ^6^, Nils Peter Petersen ^1,2^, Mia Le ^1,2,7^, Emily Victoria Nelson ^1,2^, Kouramoudou Berete ^8^, Fodé Amara Traore ^9^, Anaïs Legand ^10^, Pierre Formenty ^10^, Fara Raymond Koundouno ^4^, Alimou Camara ^3^, Stephan Günther ^1,2^, Sophie Duraffour ^1,2,†^, Sanaba Boumbaly ^3,†^

* contributed equally

^†^ contributed equally

§ corresponding author: Giuditta Annibaldis, Bernhard Nocht Institute for Tropical Medicine (BNITM), Bernhard Nocht Straße 74, 220359, Hamburg, Germany; [giuditta.annibaldis@bnitm.de](mailto:giuditta.annibaldis@bnitm.de), phone: +49 40 285380 932

**Table of Contents**

[Figure S1	2](#_Toc222316752)

[Table S1	3](#_Toc222316753)

[Table S2	4](#_Toc222316754)

[Table S3	5](#_Toc222316755)

[Table S4	6](#_Toc222316756)

# Figure S1

**Figure S1: Percent virus genome recovery based on RT-PCR Ct values.**

Percentage recovery of the Lassa virus genome as detected by nanopore sequencing, plotted against the Ct values from RT-PCR assays. Dashed lines indicate the two technical thresholds used for sequencing quality assessment: Ct > 28 and a genome recovery >50%.

# Table S1

**Lassa virus diagnostics and sequencing results of the confirmed human cases**

|  | Ct values* | | % genome recovery | | Accession number | |
| --- | --- | --- | --- | --- | --- | --- |
|  | S-segment | L-segment | S-segment | L-segment | S-segment | L-segment |
| Case 1 | 30.0 | 31.2 | 36 | 42 | PX115272 | PX115271 |
| Case 2 | 35.0 | 33.5 | - | - | NA | NA |
| Case 3 | 34.5 | 36.0 | - | - | NA | NA |
| Case 4 | 26.4 | 23.5 | 95 | 95 | PV847661 | PV847662 |
| Case 5 | 23.3 | 21.0 | 98 | 100 | PV847663 | PV847664 |
| Case 6 | 21.4 | 23.7 | 96 | 99 | PV847665 | PV847666 |

The values were used to generate Figures 2B

*Ct, cycle threshold; -, no genomic recovery; NA, not applicable

# Table S2

**Virological follow-up of Lassa fever confirmed patients by RT-PCR during hospitalization**

| Case number | Number of days post-onset | Ct value* | | RT-PCR result | Outcome |
| --- | --- | --- | --- | --- | --- |
|  |  | Ct-S | Ct-L |  |  |
| Case 1 | 4 | 30**.**0 | 31.2 | Positive |  |
|  | 13 | 34.2 | 34.4 | Positive |  |
|  | 17 | 36.3 | 35.4 | Positive |  |
|  | 20 | 45.0 | 45.0 | Negative |  |
|  | 24 | 45.0 | 45.0 | Negative | Discharged |
| Case 2 | 4 | 35.0 | 33.5 | Positive |  |
|  | 13 | 45.0 | 36.2 | Positive |  |
|  | 17 | 45.0 | 38.5 | Positive |  |
|  | 20 | 45.0 | 39.6 | Positive |  |
|  | 24 | 45.0 | 45.0 | Negative |  |
|  | 26 | 45.0 | 45.0 | Negative | Discharged |
| Case 3 | 5 | 34.5 | 36.0 | Positive |  |
|  | 13 | 36.2 | 35.9 | Positive |  |
|  | 17 | 45.0 | 45.0 | Negative |  |
|  | 24 | 45.0 | 45.0 | Negative | Discharged |
| Case 4** | 5 | 26.4 | 23.5 | Positive | Deceased |
| Case 5 | 5 | 23.3 | 21.0 | Positive |  |
|  | 13 | 31.0 | 30.5 | Positive |  |
|  | 17 | 36.3 | 35.7 | Positive |  |
|  | 20 | 36.8 | 34.0 | Positive |  |
|  | 24 | 38.3 | 45.0 | Positive |  |
|  | 32 | 36.3 | 45.0 | Positive |  |
|  | 38 | 38.7 | 45.0 | Positive |  |
|  | 41 | 45.0 | 45.0 | Negative | Discharged |
| Case 6 | 6 | 21.4 | 23.7 | Positive |  |
|  | 13 | 27.9 | 28.3 | Positive |  |
|  | 17 | 31.0 | 30.7 | Positive |  |
|  | 20 | 33.3 | 34.2 | Positive |  |
|  | 24 | 36.1 | 37.5 | Positive |  |
|  | 32 | 45.0 | 45.0 | Negative |  |
|  | 41 | 45.0 | 45.0 | Negative | Discharged |

The values were used to generate Figures 2A

*Ct of 45 indicates under the limit of detection of the RT-PCR test

**Case 4 died on 14 August 2022

# Table S3

**Single nucleotide polymorphisms (SNPs) observed on S- and L- Lassa virus RNA segments between the three nearly complete sequences obtained from human cases.**

|  | S-segment | | | L-segment | | |
| --- | --- | --- | --- | --- | --- | --- |
|  | Accession  number | GP gene | GP protein | Accession  number | L gene | L protein |
| Case 4 | PV847661 | NA | NA | PV847662 | NA | NA |
| Case 5 | PV847663 | C1411T | H471Y | PV847664 | C4942T | L1648F |
| Case 6 | PV847665 | NA | NA | PV847666 | G6115A | V2039I |

NA, not applicable

# Table S4

**Lassa virus RT-PCR results of the tissues collected from the four positive *Mastomys natalensis***

|  | Ct values* | | | | | | | | |
| --- | --- | --- | --- | --- | --- | --- | --- | --- | --- |
|  | Whole blood | | | Spleen | | Liver | | Feces | |
|  | S-segment | | L-segment | S-segment | L-segment | S-segment | L-segment | S-segment | L-segment |
| Rodent 1** | 26.0 | 19.9 | | 26.6 | 27.4 | 23.9 | 28.4 | 27.7 | 35.7 |
| Rodent 2 | 21.7 | 22.4 | | 27.0 | 28.6 | 31.4 | 29.7 | 24.6 | 27.2 |
| Rodent 3 | 37.4 | 45.0 | | 28.8 | 29.3 | 19.3 | 31.8 | 45.0 | 45.0 |
| Rodent 4 | 34.6 | 34.7 | | 26.6 | 28.7 | 32.7 | 29.9 | 29.2 | 32.0 |

*Ct, cycle threshold; Ct of 45 indicates under the limit of detection of the RT-PCR test

**Genomic recovery from Rodent 1 RNA extract was obtained with 97% of S-segment and 94% of the L-segment, accession number PV847667- PV847668.
